# Analysis and implementation of variable frequency controlled dynamic wireless charging system with half-bridge multi-leg converter topology

**DOI:** 10.1038/s41598-025-07616-z

**Published:** 2025-07-02

**Authors:** Aganti Mahesh, Bharatiraja Chokkalingam, Sanjeevikumar padmanaban, C. Santhakumar

**Affiliations:** 1 Centre for Electric Mobility, Department of Electrical and Electronics Engineering, SRM Institute of Science and Technology, Kattankulathur, 603203 Tamil Nadu India; 2 Department of Electrical Engineering, IT and Cybernetics,, University of South-Eastern Norway, Porsgrunn, Norway; 3 Department of Electrical and Electronics Engineering, K S R College of Engineering, KSR Kalvi Nagar, Tiruchengode, Tamilnadu 637215 India

**Keywords:** Wireless power transfer, Half-Bridge converter, Dynamic wireless charging, Electric vehicle, And resonant converter, Engineering, Electrical and electronic engineering

## Abstract

Resonant Inductive Power Transmission (RIPT) represents a cutting-edge Wireless Power Transfer (WPT) technology, emerging as a secure and practical solution for charging electric vehicles (EVs). While Dynamic Wireless Charging Systems (DWCS) reduce the need for large batteries compared to static charging, they entail higher initial investments. This study introduces an innovative approach to DWCS utilizing a half-bridge-based multi-legged inverter configuration. Each leg of the inverter functions independently for a transmitter coil, effectively reducing overall system costs. In this method, the Variable Frequency Control Technique (VFCT) is introduced in half-bridge DWCS with S-S and LCC-S compensations. Analyzed its VFCT on the DWCS. In addition, explores the impact of square and rectangular coils on the proposed approach, coupled with an analysis of coil gaps’ effects on receiving power. Through an exploration of the half-bridge multi-legged DWCS and a comprehensive evaluation of coil gap and size influences, this study provides valuable insights for optimizing RIPT technology to achieve efficient and cost-effective EV charging.

## Introduction

Within wireless power transfer (WPT) methodologies, Resonance Inductive Power Transfer (RIPT) uses magnetic coupling to transmit energy across an air gap and is applied in sectors like manufacturing, automation, biomedicine, material handling, and charging mobile devices and Electric Vehicles (EVs)^1,2^. This EV charging method offers a practical, secure, weather-resistant, and vandal-proof solution that integrates seamlessly into streets. It provides a safer charging experience without cords and supports various power needs, from high-power buses to low-power electric bicycles^3,4^. A significant challenge for EVs is the driving range, which traditionally requires large, expensive batteries with long charging times. Dynamic WPT (DWPT) addresses this by allowing EVs to charge while in motion, reducing battery size, and eliminating range limitations^5^. Dynamic Wireless Charging Systems (DWCS) charge EVs using over-energized magnetic couplers embedded in designated roadway sections. Key considerations for developing this technology include reliability, complexity, and costs. The optimal solution involves creating low-cost modular RIPT systems that are easy to install and maintain. Future advancements will focus on developing efficient, cost-effective large-scale RIPT roadways for on-the-move EV charging^6,7^.DEWC systems are categorized into two primary transmitter types: elongated and segmented. Elongated transmitters are cost-effective due to their single-coil large area and simpler power electronics, but they suffer from high electromagnetic interference, exposure, power losses, and low reliability. Segmented transmitters, which use multiple transmitters on the track, address some of these issues but introduce challenges in achieving a uniform power transfer profile and require more power electronic components, making the system more expensive^8,9^.

Various authors have proposed different methods concerning the size of the coil and its impact on speed. Receiver coils with a size of 1 m² have been implemented for a 2-kW charging system with a 20 cm transmission distance. Zhang et al. explored the impact of EV speed on the optimal length, concluding that the speed does not affect the optimum length when charging one vehicle per pad. The identified optimal size is 3 m, determined by the average coupling factor criteria^10^. Buja et al. computed the length of the primary coil and the spacing between primary coils using transferred energy calculations to facilitate the charge-sustaining mode for an EV^11^. The overall reliability of the system can be influenced by the length of the transmitter^12^.

**Fig. 1:**
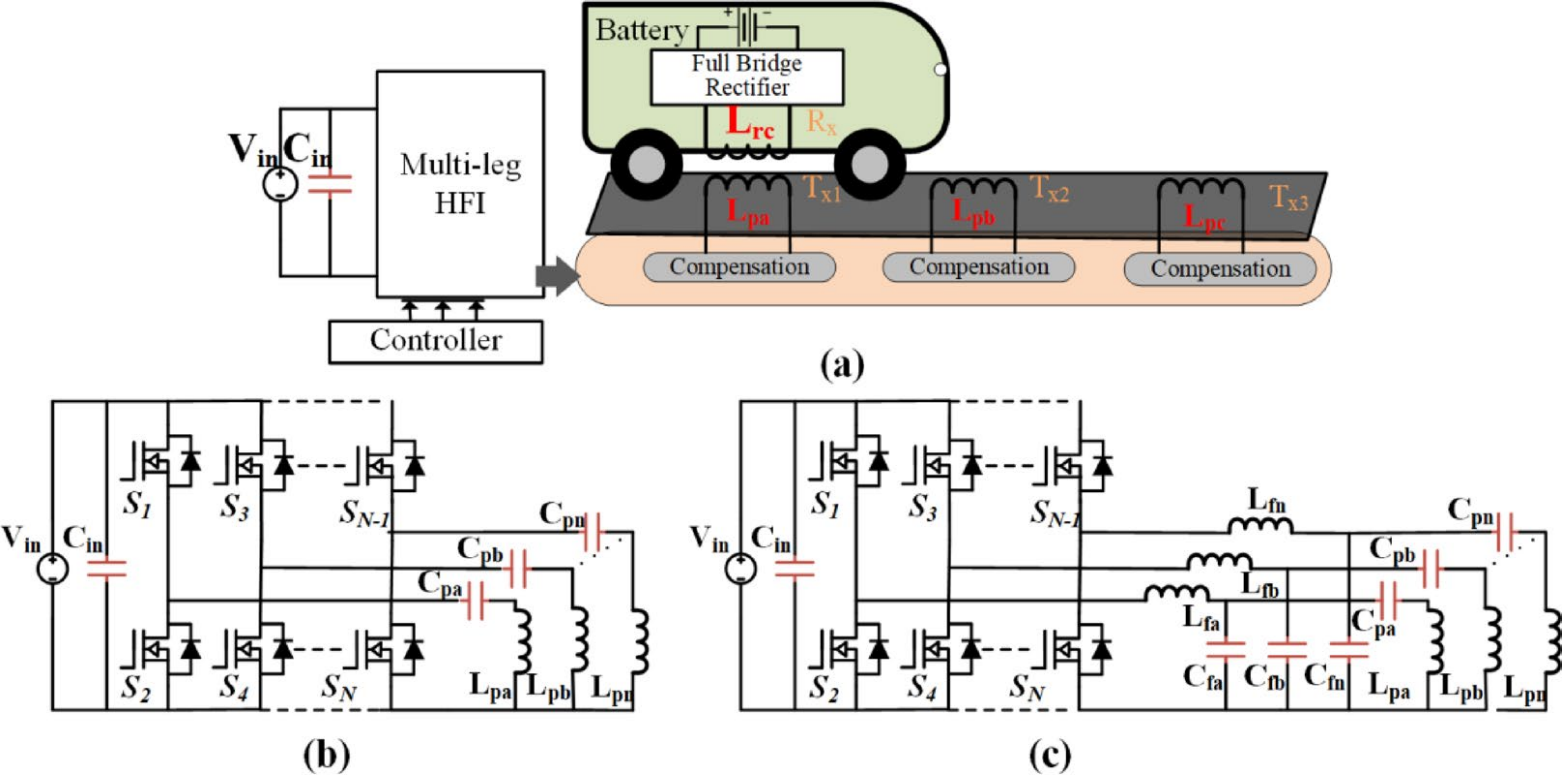
Proposed power electronic architecture for the dynamic charging system: **(a)** Dynamic charging system model, **(b)** Multi-leg converter with S-S, and **(b)** Multi-leg converter with LCC-S compensation.

For systems with a receiver coil length of less than 1 m², implementations have provided 2 kW^13^ and 26.7 kW^14^ power transmitted through an air gap of 20 cm. The use of LCC-S compensation in the transmitter and receiver setup led to a reduction in input current. In^15^a strategy for choosing the transmitter length in a long-track DEWC system is proposed, considering speed limits and energy loss. Despite addressing some challenges of elongated transmitters, segmented transmitters may encounter difficulties in achieving a uniform transferred power profile and incur higher costs due to increased power electronic components.

To meet the requirements of low-cost Dynamic RIPT systems, numerous power electronic topologies have been proposed. The system should maintain high efficiency under varying loads and incorporate a single individual full-bridge converter to a transmitter, a multi-leg converter with switching legs operating two legs as a full-bridge is proposed^16^. The drawback of the multi-converter with a full bridge is a fault in the single-leg effect of the two transmitters of the scheme. The single leg of the multi-leg converter is used as a half-bridge converter to run a separate transmitter coil to further lower the system’s cost^17,18^. In article^19^a push-pull converter-driven coupler array is proposed. Which is hard to control and coupling coefficients without severely limiting power transfer capability. A similar converter system proposed dynamic and multi-directional conversion system with a half-bridge for low power applications and with lower efficiencies. The proposed models of the dynamic charging system are depicted in Fig. 1.

The proposed topology for the DWPT system considers the effects of both series-series (S-S) and LCC-S resonance compensation topologies^20,21^. One notable distinction is observed when the system operates near the zero-phase angle (ZPA) frequency. At this frequency, S-S compensation displays characteristics of a constant current source on the secondary side, whereas LCC-S compensation exhibits characteristics of a constant voltage source on the secondary side^22,23^. This discrepancy in system behavior arises from the varying primary side resonance compensation topologies. S-S compensation is generally not well-suited for DWCS when using standard control techniques, primarily due to the significant increase in primary current under misalignment conditions. A key limitation of the S-S topology is its lack of inherent current regulation on the primary side, making it highly sensitive to variations in mutual inductance caused by vehicle movement or lateral misalignment. As a result, even slight deviations from ideal alignment can lead to substantial increases in primary current, necessitating the use of higher-rated components or the implementation of complex control strategies to limit current. In contrast, the LCC-S compensation topology naturally regulates primary current through its impedance shaping network. This feature enables more stable and predictable current behavior across a wide range of misalignment scenarios, making LCC-S a more suitable and robust choice for dynamic wireless charging environments, with reduced dependency on oversized hardware and sophisticated control mechanisms.

This study compares and analyses the distinct system features between S-S and LCC-S compensation under identical system conditions for the proposed half-bridge-based DWCS with VFCT^24^. By controlling the voltage gain with variable frequency, this technique makes S-S compensation suitable for dynamic charging systems. In dynamic EV charging, it’s unavoidable to lose soft switching, causing switch losses to vary continuously. For DEWC, power can be supplied straight to the motor, with the battery providing power only when road coupling is insufficient^25^. Preventing battery damage requires controlling bus voltage changes brought on by shifting loads or magnetic couplings. Maintaining the bus voltage at the battery’s nominal level is crucial for effective voltage control^26^. Various approaches have been suggested to achieve this.

Optimizing output voltage and transfer efficiency is achieved by using DC/DC converters on the secondary side, though they operate under hard switching conditions, reducing system efficiency^27^. Impedance matching methods also enhance efficiency. Alternatively, primary current control via Asymmetrical Pulse-Width-Modulated (APWM) switching can regulate voltage but has start-up issues^28^. APWM is simpler to design and implement but requires a broad control input range and cannot ensure Zero-Voltage Switching (ZVS) for large load variations^29^. Recently, dynamic regulators have been introduced as a solution to address the limitations of fixed-frequency WPT systems. These controllers allow the primary supply to operate at a constant frequency while minimizing losses by continuously adjusting the capacitances within the resonant loop^30^. However, this approach increases the complexity. To overcome these challenges, a variable-frequency retuned WPT system with LCL-S compensation has been presented^31^. This technique aims to improve the power transfer capability and overall system efficiency by simultaneously controlling both the frequency and duty cycle. By dynamically adjusting these parameters, the system can effectively address wide-range issues and ZVS for significant load variations^32^. This variable-frequency retuned approach has been successfully applied to static and dynamic charging systems with a full bridge converter, demonstrating its effectiveness in enhancing the performance of WPT technologies.

This article introduces VFCT to Multi-leg converter-based DWCS with a power rating of 1 kW. The outcomes were validated through both MATLAB and Ansys Maxwell simulations. A laboratory prototype was crafted to showcase practical feasibility. This paper focuses on the cost-effective DWCS approach using a multi-legged high-frequency inverter configuration, which consumes less power converters, incentive mechanisms, and adequate battery charging via sensor networks.

Our major contributions are as follows.

- A novel DWPT system utilizing VFCT is proposed, featuring a multi-legged half-bridge configuration where each inverter leg independently drives a transmitter coil, enhancing efficiency and reducing system costs.
- The effects of S-S and LCC-S compensation on the proposed DWCS were evaluated, along with an analysis of transmitter length and segment gap influence on power output.
- The impact of VFCT on S-S and LCC-S compensation was analyzed for both square and rectangular coil configurations on the dynamic charging system.

This paper is structured into multiple sections, as detailed below. Section II delves into the modeling of the proposed system, covering aspects like coil design, road line, and circuit design. Section III outlines the simulation and experimental setup of the proposed system. Subsequently, Section IV provides an analysis of the simulation and experimental results, followed by acknowledgments and the conclusion.

## Modeling of proposed system

In DWPT systems, the choice of inverter topology significantly impacts system cost, complexity, and robustness. Conventional full-bridge inverters, using two legs (four switches) per coil, provide high modularity and excellent fault isolation, as each coil operates independently. However, this approach results in a high component count and increased cost. To address this, shared-leg full-bridge multi-legged inverters reduce the number of switches by sharing one leg among coils, requiring *N* + 1 legs for N coils, nevertheless introducing challenges in control synchronization and thermal management, potentially affecting reliability. As a more balanced alternative, the half-bridge multi-legged inverter topology offers a compelling solution by requiring only N legs for N coils, with each leg functioning as a dedicated half-bridge resonant inverter. For a three-coil system, this results in just six switches, significantly reducing hardware complexity and cost. Moreover, it simplifies thermal distribution and maintains reasonable fault tolerance, making it particularly well-suited for dynamic applications like electric vehicle in-motion charging.

**Fig. 2:**
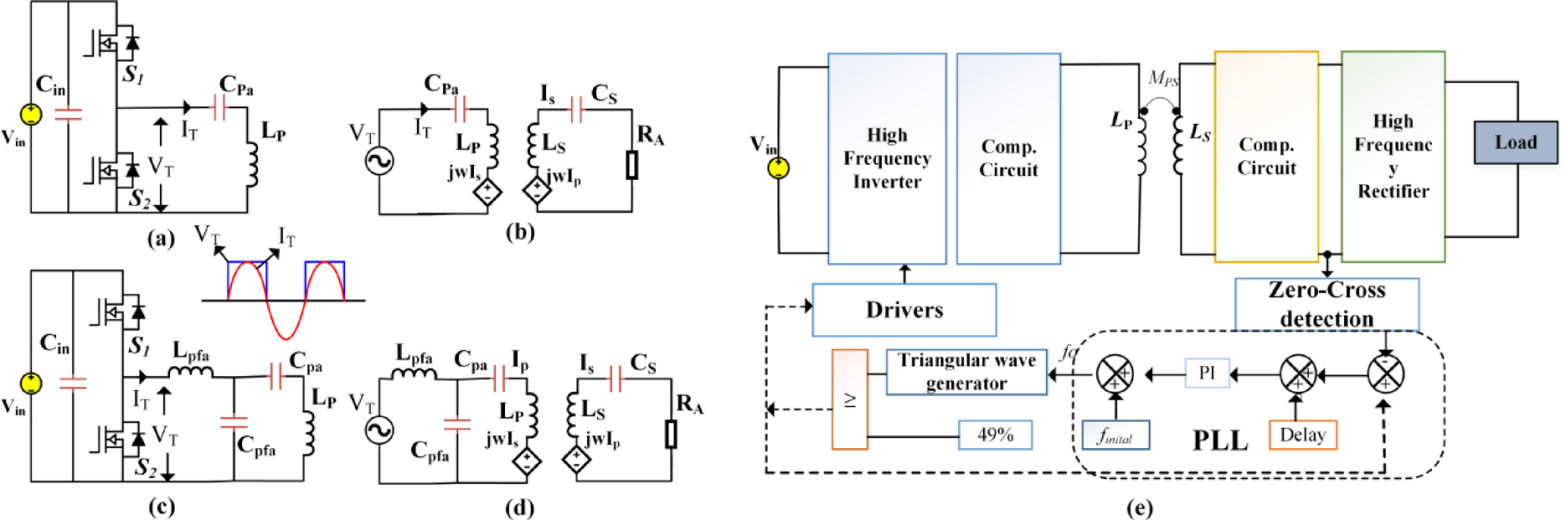
Half-bridge resonant inverter circuit with **(a)** S-S compensation, **(b)** LCC-S compensation, (c) S-S equivalent circuit, **(d)** LCC-S equivalent circuit, and **(e)** Control Scheme.

Figure 2 illustrates the proposed circuit formation when operating as a single leg. Figure 2(a) illustrates an individual leg with SS-compensation, and Fig. 2(c) presents LCC-S compensation. Their respective equivalent circuits are presented in Fig. 2(b) and 2(c). In SS compensation, C_Pa_ and C_s_ are the series compensation related to primary and secondary, respectively. Where each leg of the converter functions as a half-bridge converter. These half-bridge converters individually drive a transmitter coil, collectively powering multiple coils in a roadway system. The overall WPT system is driven by a half-bridge inverter that operates at a variable frequency (*f*_*O*_). The half-bridge inverter comprises two switches, S_1_ and S_2_, that alternately turn on and off with a dead time (*t*_*d*_) between their switching events. On the secondary side of the DWPT system, a full-bridge high-frequency diode rectifier circuit is also used to convert high-frequency AC to DC.

**Fig. 3:**
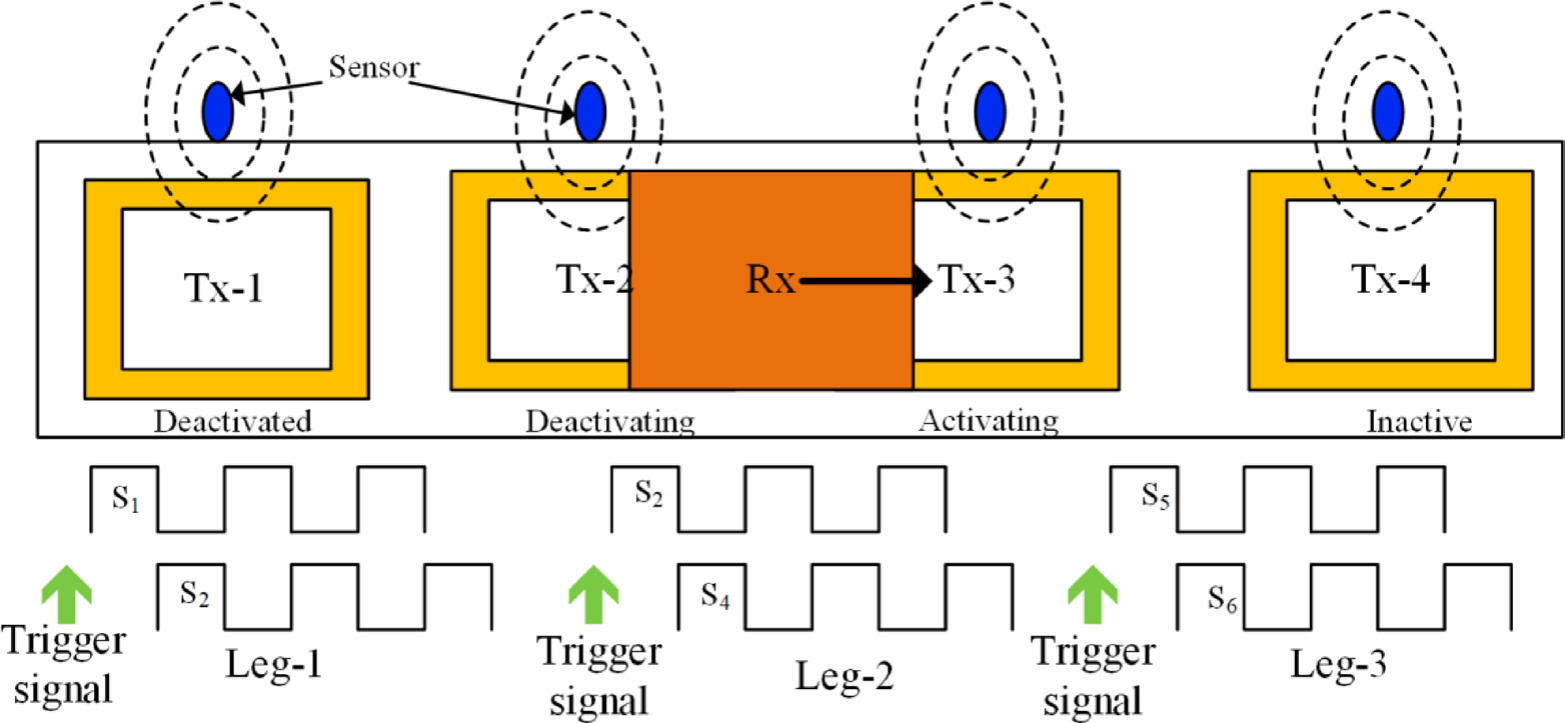
Sensor arrangement and switching pattern of the system.

Taking V_o_ as the output/battery charging voltage and P_O_ as the charging system’s output power.

The battery’s DC resistance (R_B_) can be shown as follows:

1 $$\:{R}_{B}=\frac{{{V}_{o}}^{2}}{{P}_{o}}$$.

The self-inductance of the receiver coil can be computed using the quality factor (Qs), written as

2 $$\:{Q}_{s}=\frac{{w}_{o}{L}_{s}}{{R}_{A}}$$.

Here, ω_O_ represents the resonant frequency, and R_ac_ (the equivalent AC resistance/R_A_) at the input side of the rectifier is denoted as,

3 $$\:{R}_{ac}=\frac{8}{{\pi\:}^{2}}{R}_{B}$$.

For efficient resonant wireless power transfer, the series compensation capacitance is chosen to resonate with the coil’s self-inductance at a specific frequency (Resonant frequency), enhancing energy transfer between transmitter and receiver coils. The series compensated capacitor can be,

4 $$\:{C}_{Pa}=\raisebox{1ex}{$1$}\!\left/\:\!\raisebox{-1ex}{$\sqrt{{{w}_{r}}^{2}}{L}_{P}$}\right.$$

5 $$\:{C}_{S}=\raisebox{1ex}{$1$}\!\left/\:\!\raisebox{-1ex}{$\sqrt{{{w}_{r}}^{2}}{L}_{S}$}\right.$$.

The given circuit (Fig. 2) allows representation of the WPT system using matrix form, adhering to Kirchhoff’s voltage law,

6 $$\:\left[\begin{array}{c}{V}_{T}\\\:0\end{array}\right]=\left[\begin{array}{cc}{Z}_{P}&\:-j{\omega\:}_{0}M\\\:-j{\omega\:}_{0}M&\:{Z}_{S}\end{array}\right]\left[\begin{array}{c}{I}_{T}\\\:{I}_{S}\end{array}\right]$$.

Where,

7 $$\:{Z}_{P}={R}_{P}+j{\omega\:}_{0}{L}_{P}-\raisebox{1ex}{$-j$}\!\left/\:\!\raisebox{-1ex}{${\omega\:}_{0}{C}_{Pa}$}\right.$$

8 $$\:{Z}_{S}={R}_{S}+{R}_{A}+j{\omega\:}_{0}{L}_{S}-\raisebox{1ex}{$-j$}\!\left/\:\!\raisebox{-1ex}{${\omega\:}_{0}{C}_{Pa}$}\right.$$.

Therefore, the current phasor in the Tx and Rx sides can be derived from Eq. 

9 $$\:{I}_{P}=\:\frac{{Z}_{P}{V}_{T}}{{\omega\:}_{o}^{2}{M}^{2}+{Z}_{P}{Z}_{S}}$$

10 $$\:{I}_{S}=\:\frac{-j{w}_{d}M{U}_{2}}{{\omega\:}_{o}^{2}{M}^{2}+{Z}_{P}{Z}_{S}}$$.

The AC output power can be computed as follows,

11 $$\:{P}_{out}={Z}_{P}{V}_{T}=\frac{{\omega\:}_{o}^{2}{M}^{2}{{V}_{T}}^{2}{R}_{A}}{{\omega\:}_{o}^{2}{M}^{2}+{R}_{P}({R}_{L}+{R}_{S})}$$.

Where L_P_, L_S_ are self-inductances of the primary and secondary coils, respectively. C_Pa_=C_Pb_=C_Pc_=C_Pn_ are the primary series compensated capacitances. C_S_ is series compensated secondary capacitance that is the same for SS and LCC-S compensations. Self-inductances, Primary side filter inductance, Primary side filter Capacitances, and series capacitances of LCC-S compensated ‘n’ legged system can be presented as L_Pa_=L_p.a._=L_pb_=L_pc_. =L_pn_, L_pfa_=L_pfb_=_Lpfc_…=L_pfn_, C_pfa_=C_pfb_=C_pfc_. = C_pfn_ and C_p.a._=C_pb_=C_pc_…. =C_pn_.

For the same inductance values, LCC-S compensation can be calculated from the following equations,

12 $$\:{V}_{srms}=\left|j{w}_{0}M{I}_{P}\right|=\left|j{w}_{0}M\frac{\raisebox{1ex}{$2$}\!\left/\:\!\raisebox{-1ex}{$\pi\:{V}_{in}$}\right.}{j{w}_{0}{L}_{pfa}}\right|=\left|\frac{2k\sqrt{{L}_{P}{L}_{S}}}{\pi\:{L}_{pfa}}\right|$$.

Primary side series filter inductance can be calculated from the following formula.

13 $$\:{L}_{pfa}=\sqrt{\frac{{k}_{max}{V}_{prms}{U}_{srms}}{{w}_{0}{P}_{max}}{L}_{pa}}$$.

The parallel capacitor r from the primary side calculated from,

14 $$\:{L}_{pfa}{C}_{pfa}=\frac{1}{{{w}_{0}}^{2}}$$.

Series compensation of the primary side given as,

15 $$\:{L}_{pa}-{L}_{pfa}=\frac{1}{{{w}_{0}}^{2}{C}_{pa}}$$.

As a result, two crucial IPT system characteristics are the input impedance angle and the voltage conversion ratio.

Ignoring internal resistances of the coils, the input impedance angle (θ_in_) and the conversion ratio (G_v_) of S-S and LCC-S can be derived from the following equations,

16 $$\:{\theta\:}_{in}=\frac{{180}^{0}}{\pi\:}{\text{tan}}^{-1}\frac{Im\left({Z}_{in}C\right)}{Re\left({Z}_{in}C\right)}$$.

Where C presents SS or LCC compensations input impedance^33^,

17 $$\:{G}_{V\_SS}=\frac{{V}_{o}}{{V}_{s}}=\left|\frac{jw{R}_{ac}}{{Z}_{P}{Z}_{S}+{\left(\omega\:M\right)}^{2}}\right|$$

18 $$\:{G}_{V\_LCC}=\frac{{V}_{o}}{{V}_{s}}=\left|\frac{jw{R}_{ac}}{{Z}_{in}{Z}_{S}\left[1+j\omega\:{C}_{Pa}\left(j\omega\:{C}_{P}+\frac{1}{jw{C}_{fa}}+{Z}_{r}\right)\right]}\right|$$.

This article applies a closed-loop PLL technique to the DWCS as discussed in^34^. The model diagram is presented in the Fig. 2(e). In a Dynamic Wireless Charging (DWC) system, maintaining continuous power transfer without significant dips is essential for efficient energy delivery. To achieve this, sensors are strategically placed at the midpoint of each transmitter (Tx) coil, ensuring the seamless activation of the next coil before the current one deactivates as shown in Fig. 3. For instance, when Tx1 is active, a sensor at its midpoint detects the approaching vehicle and pre-activates Tx2 before Tx1 turns off. As the vehicle moves forward, a similar process occurs with Tx2 triggering Tx3, ensuring uninterrupted power transfer. This overlapping activation method eliminates power dips, reduces misalignment sensitivity, and minimizes switching losses, thereby enhancing the system’s reliability and efficiency.

Additionally, variable frequency control is implemented using a digital Phase-Locked Loop (PLL), which aligns the phase difference between the inverter output voltage and secondary current by dynamically altering the switching frequency. The system starts at a rated frequency of 85 kHz, with the inverter operating at a 49% duty cycle to protect its switching legs. A zero-crossing detector (ZCD) senses phase differences, which are corrected using a Proportional-Integral (PI) controller, ensuring stable operation. Safety mechanisms include predefined frequency boundaries and automatic fallback to the initial frequency in case of faults or signal disruptions. These frequency limits are determined based on prior dynamic charging analysis without Variable Frequency Control (VFC), considering splitting frequencies. The rapid response of the digital PLL controller enhances adaptability, making it highly suitable for high-velocity dynamic EV charging systems by ensuring seamless and efficient power transfer across multiple transmitting coils. The equations to track the operating frequency for S-S compensation is^34^,

19 $$\:{\omega\:}_{O,180}=\sqrt{\frac{{L}_{S}{C}_{S}+{L}_{Pa}{C}_{pa}\pm\:\sqrt{{\left({L}_{S}{C}_{S}-{L}_{Pa}{C}_{pa}\right)}^{2}+4{k}^{2}{L}_{S}{C}_{S}{L}_{Pa}{C}_{pa}}}{{2L}_{S}{C}_{S}{L}_{Pa}{C}_{pa}\left(1-{k}^{2}\right)}}$$.

Further simplified to

20 $$\:{\omega\:}_{O,180}=\sqrt{\frac{1}{CL(1\pm\:k)}}$$.

As the coupling coefficient k varies due to coil misalignment during dynamic operation, the resonant frequency of the system shifts accordingly. In conventional fixed-frequency systems, this results in a mismatch between the operating and resonant frequencies, leading to reduced power transfer efficiency, increased reactive power, and unstable output. In contrast, the proposed Variable Frequency Control Technique (VFCT) dynamically adjusts the operating frequency in real-time based on system feedback to continuously track the resonant point. This allows the system to maintain optimal power transfer conditions despite variations in coupling. As a result, VFCT ensures stable and efficient power delivery while minimizing reactive losses. In systems using S-S compensation, VFCT is particularly beneficial, as it helps suppress excessive primary current rise by maintaining operation at the resonant point.

In this article, the VFCT originally applied to series-series (SS) compensation is also implemented for LCC-S compensation.

### Road line modelling

This article concentrates on a DWCS and assesses the impact of square coils with different air gap distances within it. While deploying such systems in cities hinges on factors like arterial layout, traffic patterns, and density, this study demonstrates its design through a simplified single-lane charging setup.

**Fig. 4:**
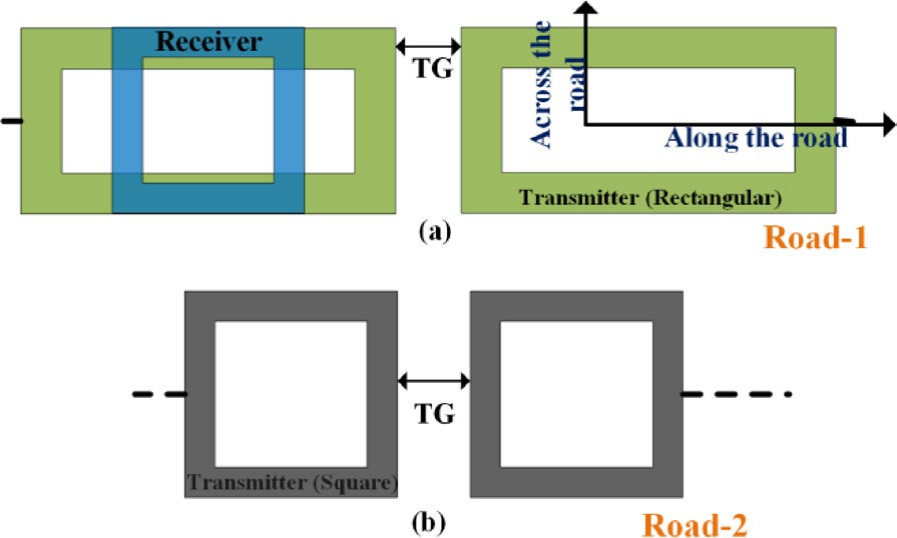
Model and movement of the receiver coil.

Two types of roadways are considered for the analysis: one with square coils and another with rectangular coils. Both cases are evaluated with two transmitter distances (TG), 0 cm and 12.5 cm. A square receiver coil is utilized for both systems. The arrangement of coils is depicted in Fig. 4, illustrating their shapes and positions. The rectangular coil length-to-width ratio has been selected as 1:2.

**Table 1:** Design parameters of the proposed System.

Parameters	Values
Power	1000 W
Frequency	85 kHz
V_o_	72 V
V_inrms_	120 V
I_prms_	8.3 A
I_srms_	15.5 A
R_L_	5.2 Ω
L_p_	109.6 µH
L_s_	31.4 µH
k	0.248
Series Compensation	
C_p_	111 nF
C_s_	33 nF
LCC-S compensation	
L_fp_	19.8 µH
C_fp_	177 nF
C_p_	39.3 nF

**Table 2:** Design parameters of the Coils.

Coil	Dimensions	No. of turns
Square Tx	25 cm x 25 cm/300 strands 38awg	24
Rectangular Tx	50 cm x 25 cm/300 strands 38awg	7.8
Square Rx	25cmx25cm/630 strands 38awg	14

Simulation and Experimental Verification.

### Simulation modeling

**Fig. 5:**
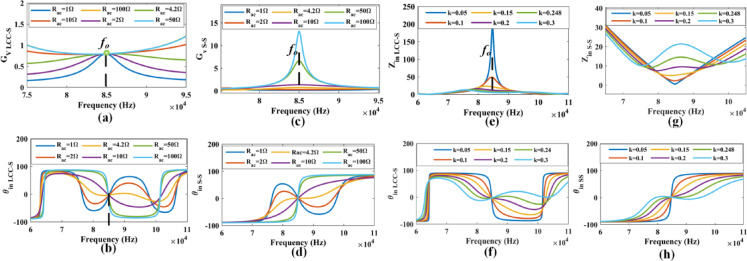
Frequency sweep effect on voltage impedance and input impedance/angle for SS and LCC-S compensations.

Table 1 provides a comprehensive overview of the design and calculated parameters necessary for the DWCS operation. These parameters were verified through simulations carried out using Ansys Maxwell and MATLAB software. The simulation outcomes are illustrated for a WPT system with a power rating of 1 kW at 85 kHz switching frequency. The effect of load resistance and coupling factor on the frequency variation is analyzed to study the effect of the frequency splitting and bifurcation phenomenon. Figure 5(a) to (b) demonstrates the impact of frequency variations on Gv and θ_in_ of the proposed system with S-S compensation for load variation depicted. Figure 5(c) to (d) demonstrates the impact of frequency variations on the Gv and θ_in_ of the proposed system with LCC-S compensation for load variation depicted. Figure 5(e) to (f) demonstrates the impact of frequency variations on the voltage gain and input phase angle of the proposed system with S-S compensation for coupling factor variation depicted. Figure 5(g) to (h) demonstrates the impact of frequency variations on Gv and θ_in_ of the proposed system with LCC-S compensation for coupling factor variation depicted.

In DWCS, the G_V_ and θ_in_ are critical parameters that determine the stability and performance of the system. The behavior of these parameters varies depending on the operating frequency and load conditions. In the S-S compensation topology, f_O_< f_L_ or f_O_> f_H_, the voltage conversion ratio G_V_ remains nearly constant despite significant variations in the load R_ac_. Near the ZPA frequency (f_O_ at 85 kHz), G_V_ increases with increasing ***R L***, reaching its maximum at the ZPA frequency. θ_in_ is zero at f_O_. When R_ac_ is large, θin is negative for f < f_O_ and positive for f > f_O_. In the LCC-S configuration, G_V_ rises with increasing frequency when R_ac_ is large. However, for a small R_ac_, G_V_ first rises and then falls as the frequency increases. Regardless of load variations, G_V_ is equal at f_O_. The input impedance angle θ_in_ is zero at f_O_, positive for f < f_O_ when R_ac_ is large, and negative for f > f_O_. Bifurcation phenomena occur when R_ac_ is small in both S-S and LCC-S configurations, generating two additional ZPA frequencies f_L_ and f_H,_ and besides f_O_. To achieve ZVS and ensure stable system operation, the system should function in the positive phase angle zone. Bifurcation commonly happens under high coupling factor k or low load resistance conditions. To avoid this, frequency control should be implemented. In DWCS, the coupling factor k is crucial. Variations in the coupling factor similarly affect voltage gain and input impedance angle as load resistance variations. The frequency splitting phenomenon occurs as the frequency deviates from the rated values, increasing with higher load resistance. By understanding the behavior of voltage conversion ratio and input impedance angle under different operating conditions, designers can optimize the performance and stability of DWCS while ensuring ZVS operation and avoiding bifurcation issues.

As vehicle speed increases, rapid changes in mutual inductance between the transmitter and receiver coils can lead to fast fluctuations in the coupling factor (*k*), potentially disrupting efficient power transfer. To address this, the proposed method incorporates adaptive modulation techniques and advanced coupling control algorithms—potentially enhanced with AI or machine learning—to maintain stable power transfer even under highly dynamic conditions such as high-speed vehicle movement. While this article does not explicitly examine the upper-speed limits of VFCT’s responsiveness to rapid inductance changes, the system is primarily designed for semi-dynamic wireless charging, where vehicle speeds are moderate and coupling variations are less abrupt. Nonetheless, VFCT’s flexible and intelligent control strategies offer strong potential for managing inductance variations at higher speeds. Although the effects of load variation are not explored in depth, VFCT demonstrates inherent robustness by dynamically adjusting the operating frequency in response to changes in reflected impedance.

Two types of transmitter coils were selected for the proposed dynamic charging system. Ansys simulation was done for a rectangular coil with different lengths. The dimensions of the first coil are 50 cm x 25 cm, while the second coil measures 25 cm x 25 cm. The receiver coil dimensions remain consistent at 25 cm x 25 cm.

**Fig. 6:**
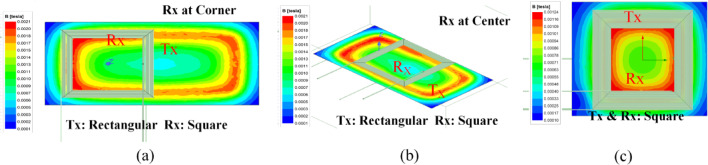
Ansys simulation: Flux density at different positions.

**Fig. 7:**
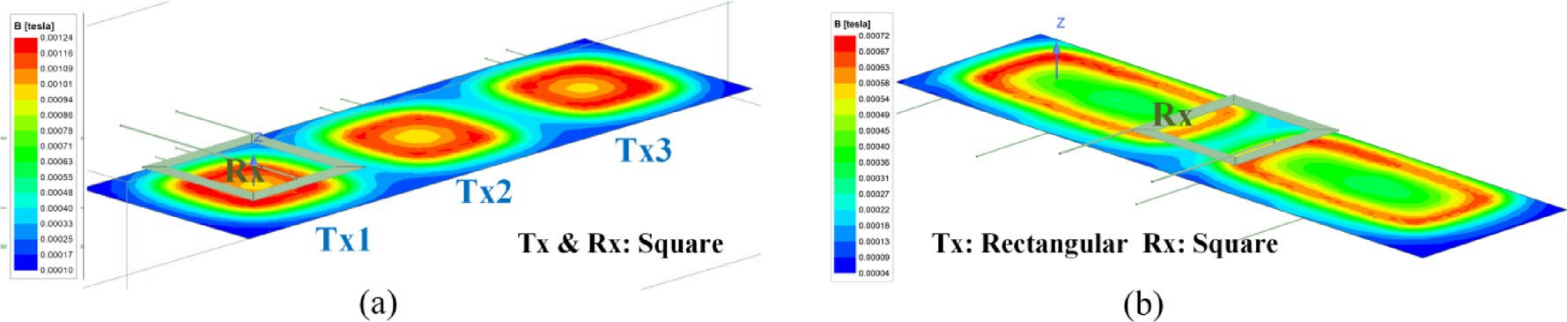
Ansys simulation: Dynamic flux analysis.

The Ansys and experimental setup are tabulated in Table 2. In Fig. 6, the flux density models Ansys Maxwell generated showcase the magnetic flux distribution within the charging system. It can be observed that the flux density is uniformly distributed across the square coil. The flux density for the rectangular coil is less than the square coil for a similar coupling factor or distance. However, rectangular coils, especially when elongated along the direction of vehicle motion (typically the longitudinal axis), offer advantages in dynamic wireless charging applications. Their extended length increases the effective coupling time as the receiver coil passes over the transmitter, thereby enhancing average output power throughout the motion. However, for the rectangular coil, the flux density is higher at the corners than at the coil’s center. That means the coupling factor between the transmitter and receiver at the center of the rectangular coil is less than the corners of the coil at the same distance. Figure 7 presents the flux distribution in dynamic conditions. Figure 7(a) demonstrates the square coil model. Figure 7(b) shows the rectangular coil model.

### Experimental setup

**Fig. 8:**
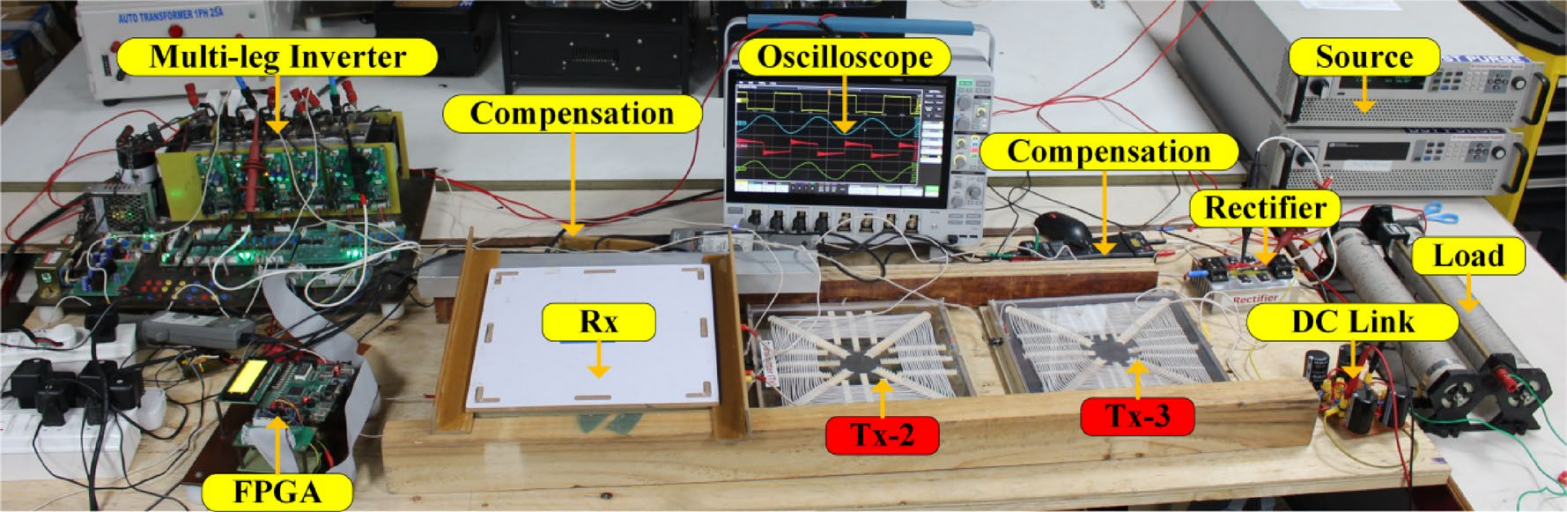
Experimental setup of the proposed system.

Following the simulation, the corresponding hardware setup was implemented, as shown in Fig. 8. The proposed method is implemented using a six-legged HFI. In this HFI, only three legs are employed to energize the three transmitter coils (square/rectangular). An FPGA Spartan 6 LX9 Micro Board gives the pulses to this system. Sensors are used to transition pulses from one leg to another leg of the inverter. RI8TU-10,016 utilized as grid side rectifier. The power transfer is achieved by the Tx coil from the HFI, which consists of six SiC power MOSFETs S1-S6 (three legs) (SCT2080KE). Subsequently, the secondary-side HF rectifier, composed of OnSemi-RURG30120, is employed to convert AC to DC for powering the equivalent load/battery.

To keep the switches on each leg from shorting out, it is expected that the signals on each pair be in the opposite phase and have very little dead time between them. A field-programmable gate array (FPGA) controls the proposed DWCS. The FPGA logic, implemented using VHDL in Xilinx ISE software, has been thoroughly validated and functionally tested. As mentioned above, the two Tx coils connected to both arms have the dimensions given in Table 2. As mentioned, the gap between the two Tx coils is kept at 0 cm and 12.5 cm. A sensor is embedded in front of each Tx coil in the FPGA logic, which acts as a trigger to track the position of the Rx coil in the wireless charging path.

**Fig. 9:**
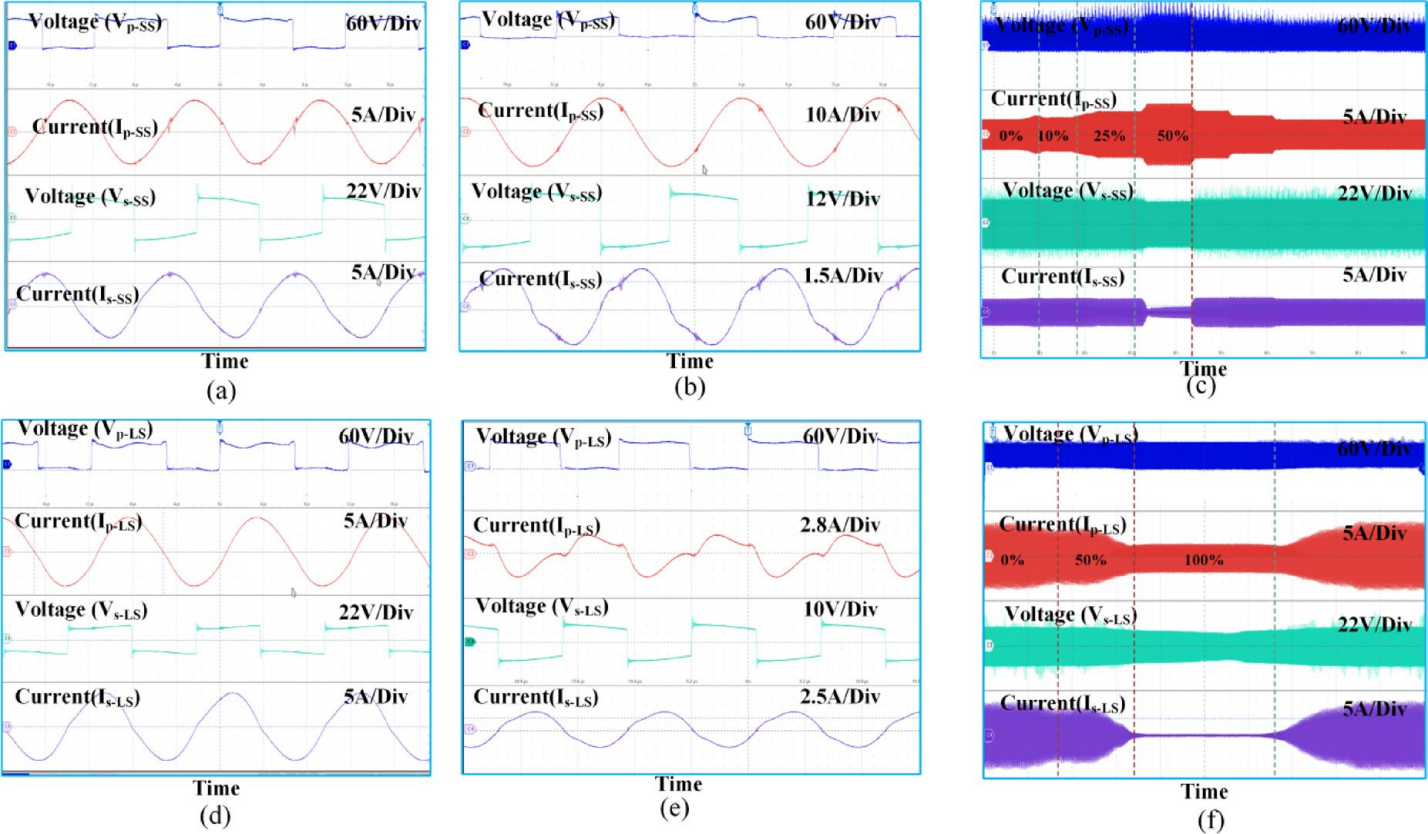
The hardware results are as follows, from top to bottom: the input voltage, followed by the input current of the primary resonant tank, the input voltage, and the input current to the bridge rectifier. **(a)** at full alignment for SS compensation, **(b)** at 50% SS compensation, **(c)** Different misalignment for SS compensation (**d**) fully alignment for LCC-S compensation, (**e**) 50% for LCC-S compensation, and **(f)** Different misalignment for LCC-S compensation (Results taken for Tx and Rx: Square).

In Fig. 9, the output voltage and current of the half-bridge inverter are displayed alongside the input voltage and current of the rectifier circuit when the Rx coil aligns perfectly with the first Tx coil and is at a 50% misalignment. Figure 9(a) and 9(b) show the inverter output voltage and current along with the rectifier circuit input voltage current of S-S compensation perfectly aligned condition and 50% misaligned condition, respectively. Vp-SS and Ip-SS indicate the inverter output voltage and current Vs-SS and Is-SS Rectifier input voltage and current of S-S compensation. Figure 9(d) and 9(e) for the LCC-S compensation, Where Vp_−LS_ and I_p−LS_ indicate Inverter output voltage and current Vs-LS and Is-LS Rectifier input voltage and current for LCC-S compensation. Observing these waveforms, the LCC-S compensation offers less ripple voltage and current compared to the SS-compensation system.

**Fig. 10:**
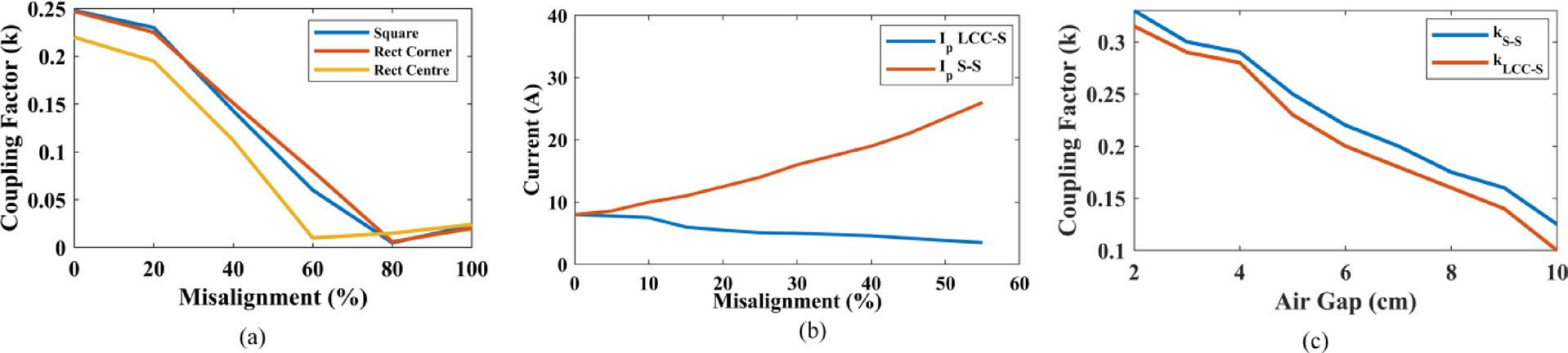
(**a**) Misalignment vs. coupling factor: Receiver coil moving across the Tx. **(b)** Change current concerning misalignment in SS vs. LCC-S compensation (Results taken for Tx and Rx: Square). **(c)** Air gap vs. coupling factor for different air gap distances.

The comparative experiment was conducted by exercising the parameters listed in Table 1 to enhance the study comparing S-S and LCC-S. Figure 9(c) and Fig. 9(f) show the change in the voltage and current waveforms amplitude magnetic coupler input and output for different misalignment positions. Figure 9(c) shows the results in the presence of S-S compensation. Figure 9(f) presents LCC-S compensation. By observing these waveforms, As the Rx coil moves away from the Tx coil, as Alignment with the coil decreases, the primary current of the S-S compensation Ip-SS Increases. With LCC-S compensation, the primary current Ip-SS decreases. These changes in the output current of the inverter concerning the change in misalignment concerning S-S and LCC-S compensation are presented in Fig. 10(b). Implementation of S-S compensation in the dynamic charging systems needs components with 5 to 6 times higher current rating LCC-S compensation or needs to implement control techniques to restrict the primary current; this makes the system more complicated. As the gap between two Tx coils increases that helps in increasing misalignment between Tx Rx the coil moving which gives more current in the primary side. Consequently, S-S systems require components; such as switches, inductors, and capacitors with much higher current ratings, increasing cost, and thermal burden. In contrast, LCC-S compensation inherently limits the primary current through its impedance-shaping network, providing more stable and predictable current behavior across misalignment scenarios. This makes it better suited for dynamic environments, LCC-S compensation is recommended in a dynamic charging system compared to S-S compensation.

Figure 10(a) shows the change in coupling factor versus misalignment for the square Tx coil and the Rectangular Tx coil with the Square Rx coil. The receiver coil at the corner position on the rectangular coil offers a higher coupling factor compared to the center of the coil at the same air gap. A square coil offers higher transmitter distance than a rectangular coil with a similar rating. The square pad offers better misalignment-tolerant behavior than the rectangular coil. Figure 10(c) presents the change in coupling factor with the air gap for square and rectangular Tx systems. By observing the figure, the square coil performs better in the vertical misalignment than the rectangular coil.

To maintain a constant output charging power, it has been demonstrated that a self-excited oscillating WPT system can deliver consistent power in the splitting region. In a DWCS, as the coils move from one transmitter coil to another, the k between the Tx and Rx varies. The maximum coupling coefficient is 0.3, while the rated coupling factor is 0.248. The coupling factor at the center of two adjacent transmitter coils depends on the sizes of the Tx and Rx coils and the distances between adjacent transmitter coils. By implementing VFCT, the system will automatically operate at the splitting frequency rather than the resonant frequency, ensuring stable operation of the proposed system.

**Fig. 11:**
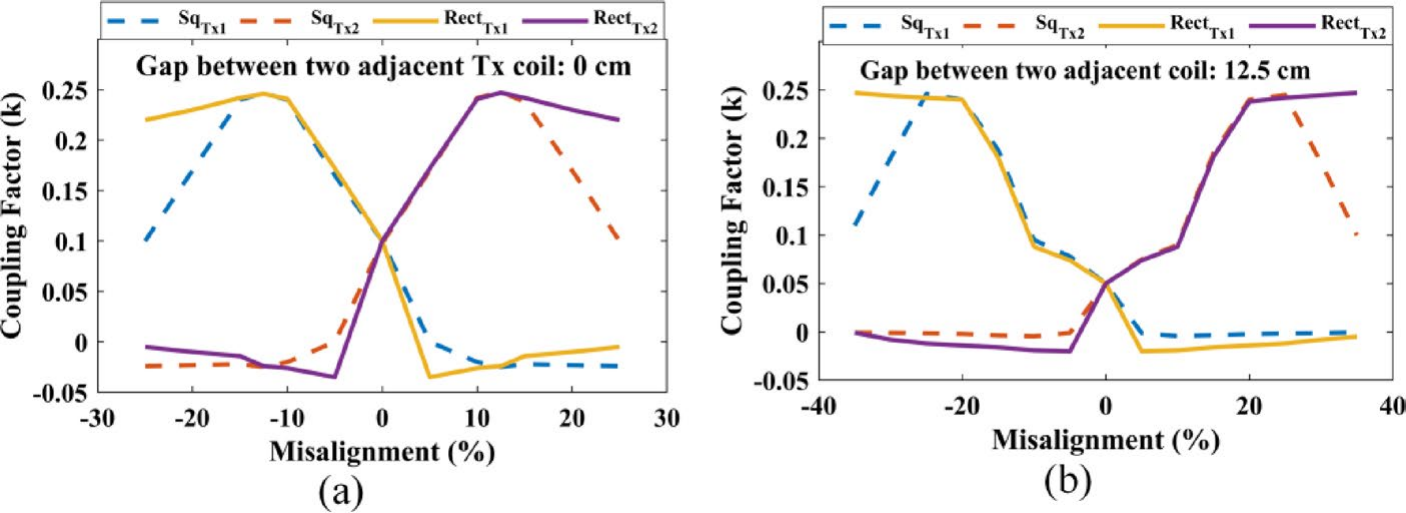
Change in Coupling Factor while transitioning between two transmitters for **(a)** 0 cm and **(b)** 12.5 cm.

Figure 11 presents the change coupling factor while the Rx coil transitions from one Tx(square/Rectangular) to another Tx(square/Rectangular) coil. When the gap between two transmitters is set to 0 cm, the lowest coupling factor is achieved at the center of the two transmitters, approximately around 0.1. Similarly, for a coil gap of 12.5 cm, the lowest coupling factor is 0.05. It is evident that as the gap between the two Tx coils increases, the lowest coupling factor also decreases. Upon examining these graphs, it becomes apparent that at peak times, the square coil transmitter coil provides a better coupling factor.

**Fig. 12:**
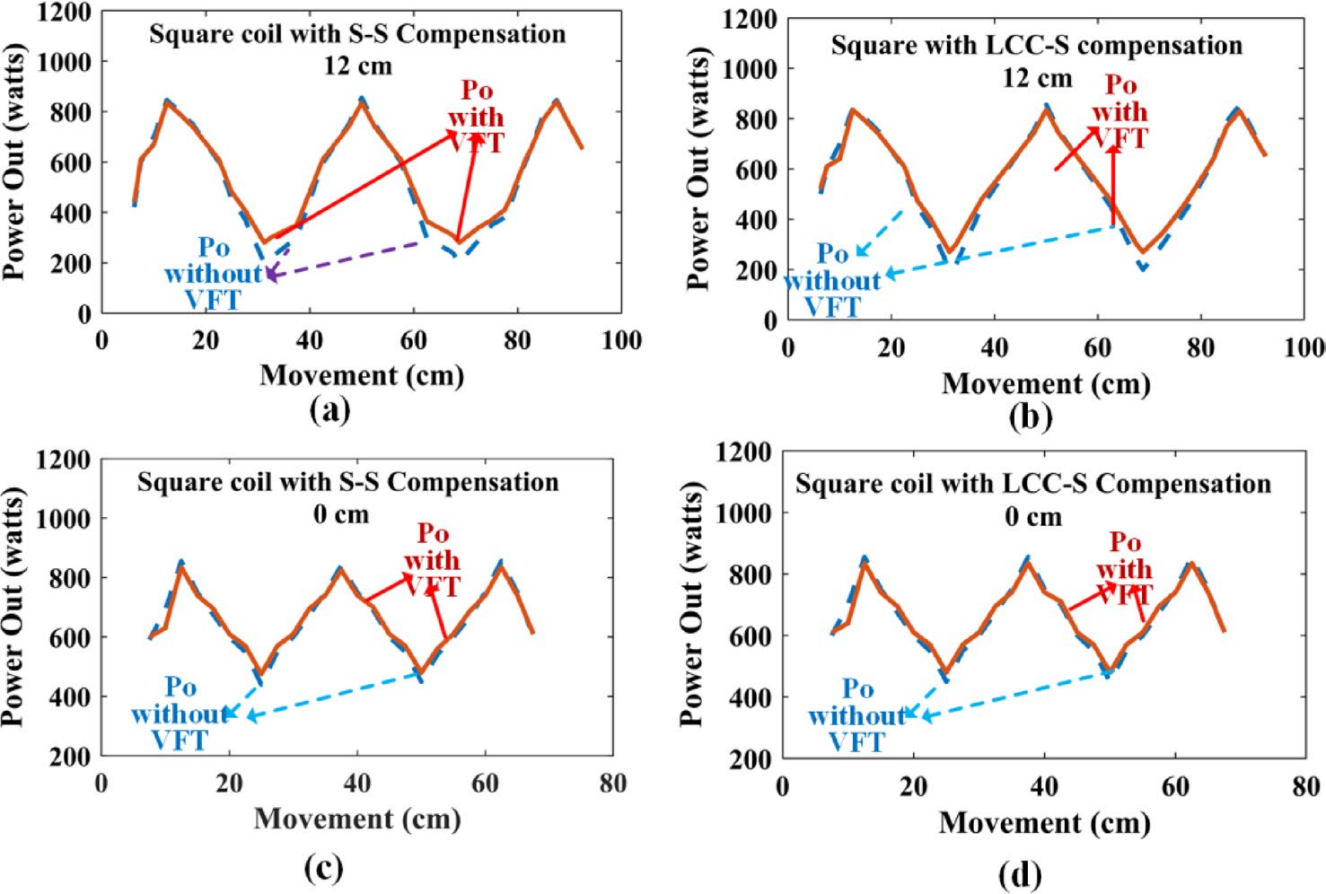
Power Out of Square Road with and without VFT technique: **(a)** 12.5 cm with S-S compensation, **(b)** 12.5 cm with LCC-S compensation, **(c)** 0 cm with S-S compensation, and **(d)** 0 cm with LCC-S compensation.

Figure 12 depicts the output power of the proposed DWCS for the square coil. With proposed method offers better average power than the general method. For a 12.5 cm gap with S-S compensation, average power increases from 490 Watts to 565 Watts. For LCC-S, increase 560 to 575 Watts. For a 0 cm gap with S-S compensation, average power increases from 530 to 565 Watts. For LCC-S increases from 650 Watts to 665 Watts. As the gap between coils is the effect of the proposed method, the effect of a zero gap between the adjacent coils is a little less. When the proposed method is applied to the LCC-S compensation, it increases the power rating; nonetheless, not as much compared to SS compensation.

**Fig. 13:**
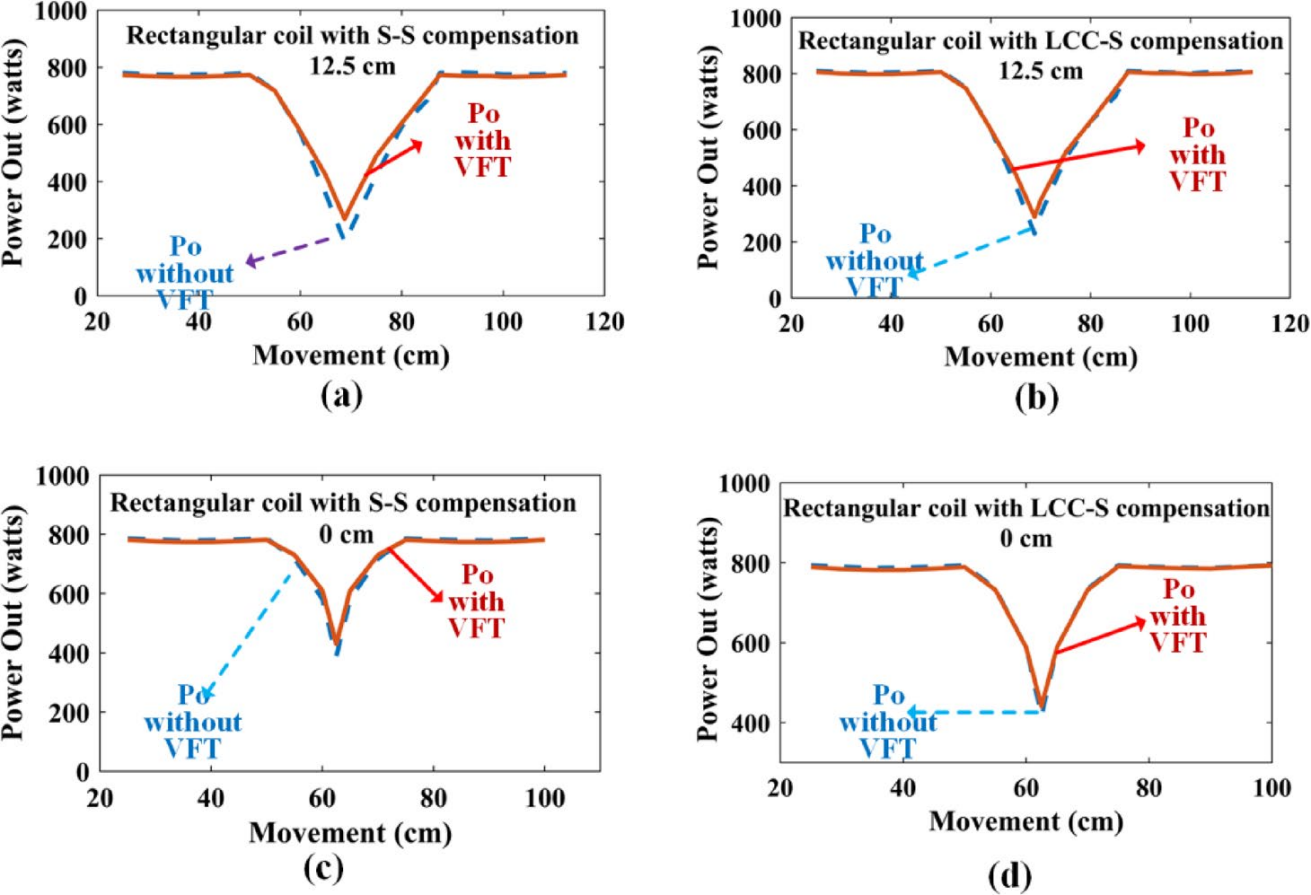
Power Out of Rectangular Road with and without VFT technique: **(a)** 12.5 cm with S-S compensation, **(b)** 12.5 cm with LCC-S compensation, **(c)** 0 cm with S-S compensation, and **(d)** 0 cm with LCC-S compensation.

Figure 13 depicts the output power of the proposed DWCS for the Rectangular coil. With proposed VFT offers better average power than the general method. For a 12.5 cm gap with S-S compensation, the power increases from 660 Watts to 670 Watts. For LCC-S, increase 695 to 700 Watts. For a 0 cm gap with S-S compensation, average power increases from 720 to 730 Watts. For LCC-S increase is from 740 to 755 Watts. As the gap between coils increases, the effect of the proposed method is at zero gap between adjacent coils is a little less. When using the recommended approach for the LCC-S compensation, it increases the power rating; nevertheless, not as much compared to SS compensation.

**Fig. 14:**
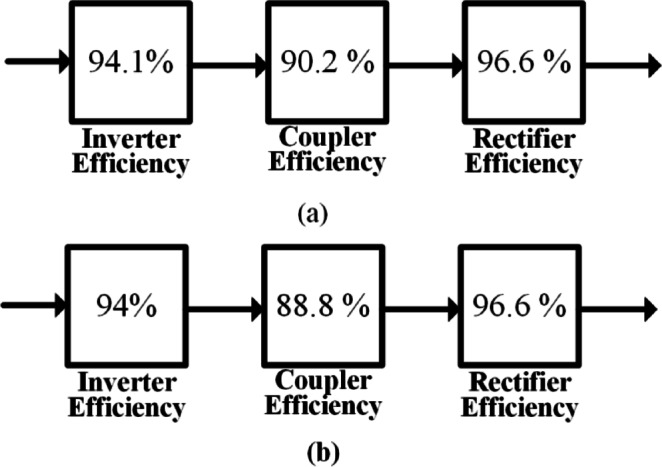
Loss distribution in the proposed system: (**a**) Square coil, (**b**) Rectangular coil.

Upon observing the graph, it becomes evident that the Square coil configuration offers higher peak power during the transition of the Rx coil from one coil to another coil. However, if considering the N-number of coils, the power fluctuations will be large. This affects the battery of the system. It is well known that the power transfer between the Tx and the dynamic system is maximized when the coils are fully aligned. Conversely, the power received is significantly lower when the Rx coil is positioned in the middle of the coil. Figure 14 presents a block diagram comparing the efficiency of dynamic wireless charging systems using square and rectangular coil configurations. As shown in the figure, rectangular coils may exhibit slightly lower peak efficiency compared to square coils due to their broader but less concentrated magnetic field distribution. However, in dynamic scenarios, they often achieve higher average power transfer efficiency, as the extended coupling duration leads to more stable and consistent power delivery.

For the square coil configuration, the inverter efficiency is 94.1%, the coupler efficiency is 90.2%, and the rectifier efficiency is 96.6%, resulting in an overall efficiency of 82.1%. In contrast, the rectangular coil configuration has an inverter efficiency of 94%, coupler efficiency of 88.8%, and rectifier efficiency of 96.6%, leading to an overall efficiency of 80.6%. The lower coupler efficiency (88.8% vs. 90.2%) contributes to the slightly reduced overall efficiency of the rectangular coil; however, it is often preferred in dynamic wireless charging systems due to its superior coupling over longer distances and greater suitability for road infrastructure integration. Despite its marginally lower efficiency, the rectangular coil provides better average output power, thanks to its extended coupling zone and enhanced magnetic field distribution along the charging path, making it an optimal choice for dynamic charging applications. Therefore, square coils are preferable in static or quasi-static applications where alignment can be closely controlled, and maximum instantaneous efficiency is the priority. In contrast, rectangular coils are better suited for dynamic charging environments, such as electric vehicles in motion, where the goal is to maximize average power transfer and maintain robust coupling over a longer track. Table 3 presents a comparison of the dynamic wireless charging system using square and rectangular coil configurations with Variable Frequency Tuning (VFT) at a 0 cm gap between two adjacent coils.

**Table 3:** Design parameters of the proposed System.

Parameters	Square Coil	Rectangular Coil
**Dimensions**	25 cm x 25 cm	50 cm x 25 cm
**Efficiency**	82%	80%
**Average Output Power**	Lower due to smaller coupling area (730 Watts approximately)	Higher due to the extended coupling zone (610 Watts approximately)

The proposed multi-leg converter topology reduces overall system cost by utilizing a shared inverter structure, where each leg independently drives a single coil in the wireless power transfer (WPT) system. This contrasts with fully modular systems, in which each transmitting coil is powered by its dedicated converter. While modular architectures offer advantages such as high reliability, simplified control, and electrical isolation, they incur higher costs and require more space due to the inclusion of complete power stages in each module.

In a multi-leg full-bridge configuration, although higher power delivery is achievable compared to half-bridge setups, the system becomes less reliable under fault conditions. A failure in one leg can affect the performance of the other legs because of the shared power and control infrastructure. Additionally, precise synchronization is required across all legs to avoid interference, increasing control complexity.

In contrast, the proposed multi-leg topology, based on individual half-bridge converters for each leg, provides a more balanced trade-off. Each leg has a simpler structure, is easier to control, and can be independently replaced at a lower cost if a fault occurs. While it may not support power levels as high as a full-bridge configuration, it offers improved cost-effectiveness, maintainability, and moderate scalability. It also retains partial modularity, making it well-suited for medium-power applications without the overhead of fully modular designs.

Overall, the proposed approach presents a practical compromise, enhancing cost efficiency and serviceability compared to modular systems, while reducing the complexity and fault sensitivity typically associated with full-bridge multi-leg topologies.

## Conclusion

This article delves into the intricacies of designing and analyzing a DWCS, with a particular focus on the effects of a half-bridge power converter on coil and compensation configurations using a variable frequency method. The research contrasts the impacts of VFT on S-S and LCC-S compensations with square and rectangular coil models. Results indicate that S-S compensation requires higher-rated components and specialized current control due to increased input current, while LCC-S compensation is better suited to dynamic charging setups. The proposed method reduces the rise in the primary current of S-S compensation by regulating voltage gain, making it more suitable for S-S compared to LCC-S compensation.

The square coil configuration exhibits 82% efficiency; however, its overall efficiency drops compared to the rectangular coil’s configuration due to more transmitter gaps, with the rectangular coil achieving a peak of 80% efficiency. Additionally, elongating the rectangular coil while keeping the width constant diminishes efficiency and power transfer distance. Notably, this article highlights that operating a single coil with a single leg, instead of using a single coil to operate with legs (Full-Bridge), improves overall efficiency. This approach could also mitigate efficiency issues and power fluctuations, warranting further exploration.

## Data Availability

The datasets used and/or analyzed during the current study are available from the corresponding author on reasonable request.
